# Exploring Factors Affecting Patient–Provider Interactions and Healthcare Engagement Among a Diverse Sample of Women Who Have Sex with Women in New York City

**DOI:** 10.1007/s10508-022-02478-2

**Published:** 2022-12-07

**Authors:** Musarrat Rahman, Rachel A. Fikslin, Eugene Matthews, Maria A. Vazquez Sanchez, Geunsaeng Olivia Ahn, Paul H. Kobrak, Elizabeth S. Lovinger, Sarit A. Golub

**Affiliations:** 1 New York City Department of Health and Mental Hygiene, 42-09 28th Street, Long Island City, NY 11101 USA; 2 Department of Psychology, Hunter College of the City University of New York, New York, NY USA; 3 Basic and Applied Social Psychology (BASP) PhD Program, Department of Psychology, Graduate Center of the City University of New York, New York, NY USA; 4 Hunter Alliance for Research and Translation (HART), New York, NY USA; 5 Columbia University Mailman School of Public Health, New York, NY USA; 6 Treatment Action Group, Washington, DC USA

**Keywords:** Women, Sexual orientation, Race, LGBTQ health, Health engagement, Patient–provider interaction

## Abstract

Women who have sex with women (WSW) have lower rates of engagement in health care and preventive screenings than women who have sex exclusively with men. Existing literature provides limited insight into how intersecting and overlapping identities, such as race, ethnicity, sexual orientation, gender identity, and identities related to gender expression, may shape individuals’ experiences within health care. We conducted qualitative interviews in New York City with 30 people who identified as women, reported sex with people who identify as women, were age 18–65, and were diverse in race, ethnicity, and sexual orientation and gender identity. The semi-structured questionnaire asked participants about positive and negative healthcare experiences to elicit what could encourage or prevent seeking care, with a focus on provider-related factors. Factors that led to positive healthcare experiences included having a provider who was knowledgeable about LGBTQ experience and health and who affirmed their sexuality, gender identity, and other intersecting identities. Factors that contributed to negative healthcare experiences included poor interactions with providers, and providers’ perceived heteronormativity and lack of awareness of WSW healthcare needs. WSW of different races, ethnicities, sexual orientations, and gender identities seek validating healthcare experiences that acknowledge and affirm their identities. We present a visual summary of the main thematic factors that contributed to positive and negative WSW healthcare experiences. Increasing access to care requires training providers on how to engage WSW patients, including WSW of diverse race/ethnicity and gender identity and expression.

## Introduction

Compared to women who exclusively have sex with men, women who have sex with women (WSW) have lower engagement in health care and preventive screening, making them more susceptible to negative health outcomes (Agénor et al., 2020; Brenick et al., 2017; Kerker et al., 2006; Knight & Jarrett, 2017; Li et al., 2015). The term “WSW” was first used in literature shortly after the term “MSM” was used in 1990 to refer to HIV/AIDS transmission risk factors, and so the introduction of the term “WSW” characterized sexual behavior as more than just “gay,” the term earlier medical literature used to refer to risk factors for HIV/AIDS transmission (Young & Meyer, 2005). An abundance of research has shown that cisgender lesbian and bisexual women are at a greater risk than cisgender heterosexual women for health problems such as cervical cancer, as well as higher prevalence of binge drinking, substance use, and tobacco smoking (Baldwin et al., 2017; Herrick et al., 2010; Kerker et al., 2006; Knight & Jarrett, 2017; New York City Department of Health and Mental Hygiene, 2017; Pharr et al., 2019; Trinh et al., 2017). Compared to women who exclusively have sex with cisgender men, WSW have greater diagnosis of bacterial vaginosis and sexually transmitted infections (Knight & Jarrett, 2017; New York City Department of Health and Mental Hygiene, 2017), as well as mental health issues including depression, anxiety, and suicidal ideation (Knight & Jarrett, 2017; New York City Department of Health and Mental Hygiene, 2017).

Heterosexist assumptions on the part of providers can contribute to health inequities for WSW (e.g., Tabaac et al., 2019; Wingo et al., 2018). For example, higher prevalence of cervical cancer was linked to a lack of provider education, providers’ false belief that WSW are not at risk of the human papillomavirus (HPV) and cervical cancer, and less engagement in care due to poor experiences with providers due to stigma and medical mistrust (Brenick et al., 2017; Brown & Tracy, 2008; Cochran et al., 2016; Henderson, 2009). Existing research on women of color has found that among lesbian and bisexual women, Black women are at a greater risk of cervical cancer than White women (Agénor et al., 2015; Durso & Meyer, 2013; Molina et al., 2014). Additionally, neglecting to ask patients about the genders of their sexual partners can lead providers to make incorrect assumptions about sexual behaviors (Baldwin et al., 2017; Howard et al., 2019; Venetis et al., 2017), which may result in misguided clinical recommendations.

Most research on facilitators and barriers of care among WSW focuses on the experiences of White, cisgender women who identify as lesbian or bisexual, and seldom involves intentional recruitment of participants of diverse races, ethnicities, sexual orientations, and gender identities amongst WSW. Furthermore, despite known health inequities based on race (Agénor et al., 2015; Durso & Meyer, 2013; Mays et al., 2002; Molina et al., 2014), research on WSW seldom addresses the intersecting and overlapping systems of oppression that women of color experience within health care.

To understand the health experiences of WSW, data collection must consider overlapping and intersecting social identities. Intersectionality theory is a framework developed by Black feminist scholar Kimberlé Crenshaw (e.g., Collins, 1990; Crenshaw, 1990; Taylor, 2017) that conceptualizes that each person has different overlapping and intersecting social identities, and can experience multiple systems of oppression and disadvantage based on hierarchies of power, e.g., race, ethnicity, sexual orientation, gender identity, economic background, religion, ability, or all of them simultaneously (Bauer, 2014; Bowleg, 2008; Crenshaw, 1990). In recent years, there has been an increased call for research that approaches LGBTQ health through an intersectional perspective to better understand the impact of racism and other systems of oppression on underrepresented perspectives and voices within our data (Chan & Henesy, 2018; Corus & Saatcioglu, 2015).

Research on patient clinical experiences that includes Black, Latina, Asian/Pacific Islander WSW highlights the presence of racial discrimination in the clinic and discomfort with providers due to the lack of providers who share their races and ethnicities (Agénor et al., 2015; Brenick et al., 2017; Li et al., 2015). In one study, Asian/Pacific Islander and Latina women who identify as queer (an umbrella term used to describe sexual and gender identities beyond heterosexual and cisgender) emphasized the importance of having a provider of the same race and ethnicity who was respectful of their sexuality and understanding of the ethnic context due to cultural stigma of being a WSW within their communities (LaVaccare et al., 2018). Queer women of color often prefer to have providers of color as well as providers who are LGBTQ or LGBTQ-knowledgeable (Agénor et al., 2015; Baldwin et al., 2017; Hiestand et al., 2007; Howard et al., 2019; LaVaccare et al., 2018). These preferences may be rooted in attempts to avoid discrimination and build stronger relationships with healthcare providers (Agénor et al., 2015; Baldwin et al., 2017; Hiestand et al., 2007; Howard et al., 2019; LaVaccare et al., 2018).

Historical race-based medical malpractice and personal experiences of racism can lead to medical mistrust among Black women and undermine engagement in care (Brenick et al., 2017; Li et al., 2015). Black women have described how medical providers can make assumptions about their social and economic status based solely on their race (Agénor et al., 2015). Additionally, one study demonstrated that for Black WSW the experience of stigma related to sexual orientation in any setting was associated with lower engagement in care and led to less identity disclosure in healthcare settings (Brenick et al., 2017).

In most research, the term “women” has been limited to those assigned female at birth and who identify their current gender identity as a woman. Most studies on WSW do not adequately consider gender diversity, leading to a large gap in the knowledge of healthcare experiences of transgender, non-binary, and genderqueer individuals who may also hold an identity as women and as WSW. Transgender women are more likely than cisgender women to experience persistent discrimination and mistreatment in healthcare settings, including incompetent care, delay of care, and complete denial of care (Glick et al., 2018; Howard et al., 2019; Macapagal et al., 2016). Transgender women and non-binary people of color who seek care at LGBTQ-knowledgeable health centers still fear transphobia or racism in those settings (Howard et al., 2019).

Limited research considers how gender expression relates to healthcare experiences. One study found that people who identify as women or were assigned female sex at birth who identify their gender expression as butch (i.e., masculine-presenting) are more likely than femme-identified women (i.e., who present as feminine) to be open about their sexuality in healthcare settings, to actively seek out LGBTQ-knowledgeable care, and to perceive poorer treatment (Hiestand et al., 2007). Additional research is needed that explores the impact of gender expression on care experiences and engagement, especially within the context of intersecting social locations and identities.

Trauma is defined as an emotional response to an experience that is emotionally stressful and shocking (Alessi & Martin, 2017). Existing research has demonstrated that lesbian and bisexual women have a greater exposure to potentially traumatic events than heterosexual women (Roberts et al., 2010). Furthermore, bisexual, and other non-monosexual women are at higher risk of experiencing intimate partner violence than heterosexual and lesbian women (Coston, 2021). Experiences of trauma both in health care and outside of health care can affect the needs and experiences of patients in care (e.g., Palmieri & Valentine, 2021). Trauma-informed care is an affirming approach to care that both respects and acknowledges the impact of trauma on wellbeing on communities impacted by racism and other systems of oppression (Coston, 2021; Raja et al., 2015). Minority stress theory describes how racial, ethnic, and sexual minorities experience a continuum of stressors from experiences of discrimination and stigma, which can be considered traumatic experiences (Meyer, 2003). Considering a trauma-informed approach is essential when taking an intersectional perspective because overlapping systems of oppression contribute to the prevalence of traumatic experiences due to stigma and discrimination or due to other forms of trauma, such as sexual assault (e.g., Coulter et al., 2017).

Research on the healthcare experiences of WSW is needed to identify structural drivers of inequities they face receiving preventive health care and engaging in continuous care (Brenick et al., 2017; Glick et al., 2018; Kerker et al., 2006; Li et al., 2015; Macapagal et al., 2016; Tabaac et al., 2015). In addition to the ways racial and gender experiences can intersect, we have limited understanding of how age can further affect experiences of care, particularly for older WSW and healthcare utilization and engagement. Methods that consider multiple identities and bases of experience, including age, can expand our understanding of the complex experiences of diverse WSW.

The current study aimed to explore (1) barriers and facilitators to health care in a diverse sample of WSW and (2) how intersectional and overlapping identities may influence the quality of care and level of engagement for WSW within health care.

## Method

### Participants

We recruited participants by posting digital flyers on the social media platform Facebook as well as through digital and physical outreach at community-based organizations, pride events, and at LGBTQ community centers, bars, and bookstores across New York City. We then screened interested participants over the phone to determine eligibility and to assess demographics to ensure diversity across racial, ethnic, and sexual identities. Participants were eligible if they spoke English, were between the ages of 18 and 65 years, and identified as a WSW. In order to include a range of experiences that capture the gender diversity among women have sex with women, we used self-identification as the inclusion criteria, such that anyone who identified as a woman who has sex with women was eligible for the study, whether they were cisgender, transgender, or non-binary. Institutional Review Boards for the New York City Department of Health and Mental Hygiene (DOHMH) and Hunter College of the City University of New York (CUNY Hunter) reviewed and approved the study prior to study procedures. All participants provided informed consent before interviews were conducted. We compensated participants US $40 in cash for their time.

The 30 participants ranged from 18 to 58 years of age with a median age of 30 years (see Table 1). We used multiple choice questions with the option to “select all that apply” to assess participant demographics. Participants identified as multiracial (23%) or exclusively as White (40%), Black/African American (20%), or Asian/Pacific Islander (13%), while 3% declined to respond. Seventeen percent identified as Latina, most of whom also identified as multiracial. Cisgender women made up 83% of the sample, followed by 13% who identified as both non-binary and women, and 3% identified as transgender women. In terms of sexual orientation, 37% identified exclusively as lesbian and 26% as multiple sexual orientations. All the individuals who selected multiple sexual orientations selected queer as one of their identities. Fifty percent of the sample were on Medicaid, 50% reported an annual household income of less than $20,000, and 36% were unemployed (of whom 82% were people of color). In terms of recruitment, 50% of the sample found out about the study through Facebook.

**Table 1:** Participant characteristics

Characteristics	N	%
*Age in Years* Median, Mean (Range)	30, 35 (40)	18–58
*Race*		
Asian/Pacific Islander	4	13.3%
Black/African American	6	20.0%
Middle Eastern or North African (MENA) ^a^	1	3.3%
Multiracial	7	23.3%
White	12	40.0%
Declined to respond	1	3.3%
*Ethnicity*		
Latino/a (of any race)	5	16.7%
*Sexual Orientation*^b^		
Asexual	2	6.7%
Bisexual	7	23.3%
Lesbian	14	46.7%
Pansexual	4	13.3%
Queer	13	43.3%
Sapiosexual^c^	1	3.3%
Not Straight	1	3.3%
More than one of these categories	8	26.7%
*Gender Identity*		
Cisgender Woman	25	83.3%
Nonbinary and Woman	4	13.3%
Transgender Woman	1	3.3%
*Type of Health Insurance*		
Medicaid	15	50.0%
Private	14	46.7%
None	1	3.3%
*Annual Household income*		
Less than $15,000	11	36.7%
$15,000—$19,999	4	13.3%
$20,000 -$29,999	7	23.3%
$30,000 -$69,999	5	16.7%
$70,000 and above	3	10.0%
*Response to: How did you find out about this study?*		
Facebook	15	50.0%
Through a friend	6	20.0%
Community-based Organization	7	23.3%
Brooklyn Pride	2	6.7%

^a^Participant reported being both MENA and white (but did not identify as multiracial) and is also counted under the Multiracial category

^b^Participants were allowed to choose more than one sexual orientation. Of these, eight participants (26.7%) reported more than one sexual orientation. The totals and percentages here total more than 100%

^c^Sapiosexual means that one is sexually attracted to or aroused by intelligence

### Measures and Procedure

We conducted semi-structured interviews with 30 WSW between June and August of 2018. Eligible participants were given the option to schedule an in-person interview at one of the two study locations, either at the DOHMH in Long Island City or CUNY Hunter in the Upper East Side. Interviews lasted 60–90 min. Participants were asked about their social identities and how those identities impacted their healthcare experiences, their sexual health care, and their recommendations for improving healthcare experiences for WSW. We asked questions about positive and negative healthcare experiences, broadly defined (i.e., including in primary care, sexual health care, traditional medicine/healing, mental health, and any other specialty health care) to elicit what encourages or prevents seeking care among WSW. Recruitment was conducted until thematic saturation was reached.

We elicited participant experiences through broad, open-ended questions about healthcare experiences in general (e.g., “Generally, how would you describe the health care you receive?”) as well as about positive and negative aspects specifically (e.g., “Tell me about one of the best experiences you’ve had with a provider about your health.”; “Tell me about one of the worst experiences you’ve had with a provider about your health.”). Regarding participant identities, we first asked participants to self-generate the identities that were most important to them, then asked questions that applied to important identities as self-defined by the participant (e.g., “How do you feel that different parts of your identity shape your experience with healthcare treatment you have received?”) and questions specific to gender, sexual orientation and race and ethnicity (e.g., “Have you ever disclosed to any providers that you are (or insert participant sexual identity here)?”). Questions about sexual health care included general questions (e.g., “When was the last time you discussed your sexual health with a provider? How did that conversation go?”) and questions about specific preventative screenings (e.g., “Have you ever had a breast or chest exam from a doctor?”). Additionally, we asked about participants’ embodied experience in care (e.g., “Would you say that you like or do not like the way that medical providers talk about your anatomy and your body?”). At the end of the interview, we asked participants a few general questions about the topic of interest (e.g., “What do you think are the main challenges for women who have sex with women in their experiences of health care?”).

### Data Analysis

Interviewers received training about the main study goals, semi-structured interview techniques, standardized study protocols, and coding practices. To aid in analysis and address any differences between interviewers, each interviewer took notes after each session about main themes and completed a reflexivity exercise about any power dynamics that they felt were present in the session, including different racial, gender, socioeconomic, sexual orientation, or other differences or similarities that may have affected the comfort of the participant or the themes that the researcher perceived to be important. Each interview was audio recorded, manually transcribed using Express Scribe software and entered into ATLAS.ti qualitative analysis software for coding. During transcription, any potentially identifying information was redacted to ensure participant confidentiality in data analysis.

We used an inductive thematic analysis approach to conduct data analysis of the full interview transcripts (Alhojailan, 2012; Richards & Hemphill, 2018). We employed an inductive approach to center participant experiences and identify themes related to understanding positive and negative aspects of care experiences through an intersectional lens. Members of the research team met to develop a preliminary codebook of themes related to positive and negative healthcare experiences. To ensure that the codebook was capturing the main themes and to ensure coder reliability, four primary members of the research team (the first four authors) pilot tested the codebook on a subset of interviews. Coders met to discuss any additional themes to be added to the codebook and to resolve any discrepancies in coding. This process was repeated several times until high consistency across coders was reached, and final codebook was agreed upon (see Table 2). Each interview was coded by one of the four coders using ATLAS.ti. Finally, the team lead reviewed all the independent coding to confirm consistency across coders.

**Table 2:** Preliminary Codebook

Primary Code	Keywords	Code Definition	Examples
Positive Care	Satisfied, good, positive, most important, best, aware	Participant refers to positive experiences with health care or a healthcare provider	Insurance, technology, LGBTQ-knowledgeable care, free services, incentives
Negative Care	Difficult, negative, compromise, worst, least important, a long time, unaware, mistreated, ignored, main challenges, pain, discomfort	Participant refers to negative experiences with health care or a healthcare provider	Insurance, rushed appointments, long wait times, far facility distance, no informed consent, not LGBTQ-knowledgeable, intake forms not inclusive
*Subcode:* Discomfort/Dismissal			
Anticipated stigma			
Race/Ethnicity/ Heritage	Race, Racial, Ethnic, White, Black, Asian, Pacific Islander, Middle Eastern, Arab, Latino/a, Hispanic, Mixed race, Ashkenazi Jew, Sikh, Chinese, Punjabi	Participant refers to their race/ethnicity/heritage as a factor impacting either (1) being a WSW in the context of health or (2) their experience with health care or a healthcare provider	Coming out to family and health providers, stigma, intersectional discrimination, trauma, resilience, assumptions about sexual behavior
Sexual Orientation/ Sexual Behavior	Sexual identity, WSW, lesbian, bisexual, queer, pansexual, asexual, kinky, dyke, sapiosexual, fluid	Participant refers to their sexual orientation as a factor impacting either (1) being a WSW in the context of health or (2) their experience with health care or a healthcare provider	Coming out to family and health providers, stigma, intersectional discrimination, trauma, resilience, assumptions about sexual behavior, homophobia, biphobia
*Subcode*: Sexual Behavior		Participant refers to their sexual behavior as a factor impacting either (1) being a WSW in the context of health or (2) their experience with health care or a healthcare provider	
Additional Identities	Sex worker	Participant refers to their additional identity as a factor impacting either (1) being a WSW in the context of health or (2) their experience with health care or a healthcare provider	
Gender Identity/Gender Expression	Woman, cisgender, non-binary, transgender, female, cis, trans, masculine-of-center, feminine, gender non-conforming, binary, femme, butch, boi, stone	Participant refers to their gender identity/gender expression as a factor impacting either (1) being a WSW in the context of health or (2) their experience with health care or a healthcare provider	Coming out to family and health providers, stigma, intersectional discrimination, trauma, resilience, assumptions about sexual behavior, transphobia, gender affirmation
Recruitment	Flyer	Participant refers to what motivated them/why they decided to respond to the flyer and participate in the research study	Identity affirmation, never seen a study like this before, incentive
Health Motivation	I try to, I make sure, I seek, I feel	Participant refers to individual behaviors or attitudes toward their health and health care	Screening, medication adherence, healthcare seeking
Recommendations	Should, can, suggest, hope, wish; currently want or need	Participant offers recommendations on what providers can do to improve health care	Affirming spaces; inclusive forms; sexual history-taking
*Subcode*: Desired Care			
Access to a provider	Seek	Participant refers to factors that enable/disable them from seeking a healthcare provider	Health insurance, technology, LGBTQ-knowledgeable care, free services, incentives
*Subcode*: Provider outside of the U. S			
Identity Disclosure	Provider knows, told; disclosed; closeted	Participant discloses their sexual orientation or sexual behavior to providers	Sexual history-taking

**Fig. 1:**
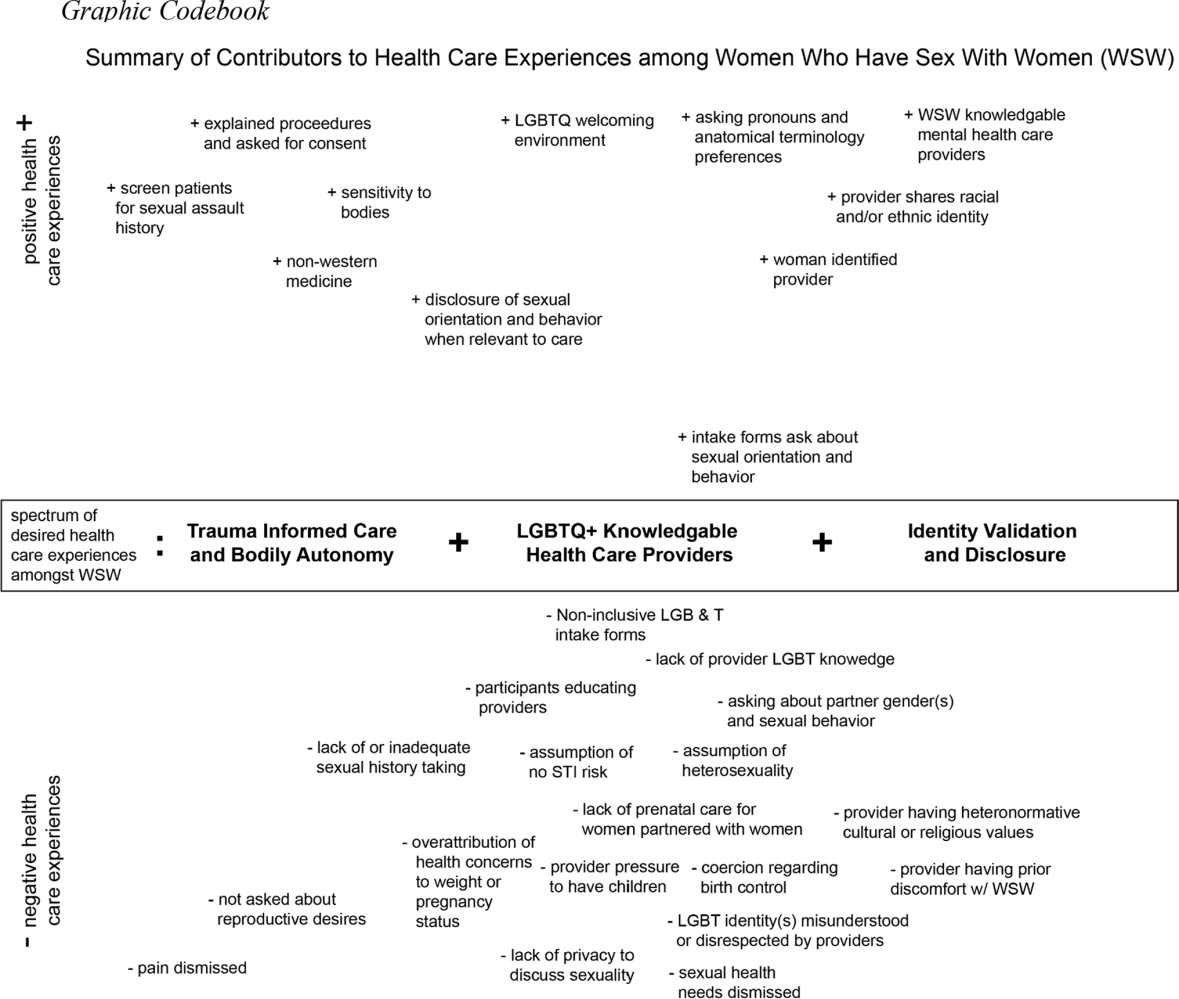
Graphic Codebook

After the main coding was complete, the team lead reviewed and organized content into sub-themes that serve as facilitators and barriers to affirming care to be presented in the results section using an Excel spreadsheet. It is worth noting that while trauma-informed care was not an explicit part of our initial codebook, the research team observed themes emerging around positive and negative patient–provider interactions related to bodily autonomy, consent, past experiences of sexual trauma, and participant self-care around traumatic experiences. The full team met several times to review and discuss how to summarize main themes and sub-themes ultimately determining that most important facilitators and barriers could be organized into the three main theme categories presented below and subdivided into positive and negative experiences of care. Quotes were selected with attention to presenting the range of experiences participants shared in relation to their racial, ethnic, sexual, gender identities and expression, age, religion, and other intersecting identities. After the results organization was solidified, one of the authors organized the main themes into the graphic codebook to present a visual depiction summarizing key findings (see Fig. 1). The research team met to review, discuss, and edit the graphic codebook until consensus was reached.

## Results

During the qualitative interviews, participants described both positive and negative experiences when receiving health care and whether they experienced any bias or negative care due to one of their identities. Analysis of interview content identified three aspects of health care particularly important to WSW: (1) identity validation and disclosure; (2) having LGBTQ-knowledgeable care providers; and (3) having providers with a trauma-informed approach to care (see Fig. 1). Participants provided insight into these three main themes about both what factors contributed to positive experiences in care (i.e., positive care) and negative experiences in care (i.e., negative care). Each of the three themes below are broken into two sections, positive care, and negative care, to demonstrate these two types of experiences. Almost all participants expressed health insurance coverage as the top facilitator of access to a provider, emphasizing the importance of context of structural and institutional barriers to accessing care.

### Identity Mapping

When asked to list important identities that shape their experience with health care and healthcare needs, White WSW tended to report their sexual orientation as an identity that shapes their care, while WSW of color were more likely to report their race, ethnicity, or heritage as an identity that shapes their care. Participant R (26 years old, Asian/Pacific Islander, multiple sexual orientations) described their identity as:

I guess the most important one, I mean right now is just being Asian, also Asian American, being Chinese. I’m teaching Asian communities of teachers like kind of intersectioning with that. I feel like my queer identity has more to do with—I guess me being transcultural.

Participant U (24 years old, White, bisexual/queer), described their identity as:

I guess largely how I identify, and […] so I have queer with bisexual overlapping along with feminist and progressive and tomboy off to the side. I have my professional identities attached to queer cause it does affect my professional life the way that I present—and then I’m a Cancer and I identify strongly with it.

### Identity Validation and Disclosure: Positive Care

Identity validation refers to experiences where a participant felt their racial, ethnic, sexual, or gender identity was affirmed by a provider. Participants highlighted experiences in which providers affirmed their authentic selves, which allowed them to feel empowered around their health care. Participants felt that having intake forms ask about sexual orientation or sexual behavior helped them to feel included and to disclose their sexuality, or “come out” to their providers. Many participants described how being asked about the kind of sex they had, and the gender of their sexual partners made them feel affirmed in their sexual identity when communicating with providers. Participants also mentioned the importance of having mental healthcare providers who knew how to support WSW, especially the challenges of being open about their sexual orientation in various social contexts as well as reconciling identities within their racial, ethnic, and queer communities.

When describing a positive experience in care, D (18 years old, Asian/Pacific Islander, lesbian) described feeling validated by their provider both on account of their race and ethnicity as well as their sexuality:

[My therapist]’s really validating of my feelings of how I feel about living a double life as this Punjabi lesbian that kind of has to be a certain way at home but trying to be myself with my friends […] I don’t know, she’s just really supportive and she’s not—she’s not weird about me being gay or anything.

Various WSW of color expressed the importance of having a provider of color or of the same racial and ethnic identity, who is LGBTQ, and who is a woman. Participants were able to connect with and feel more comfortable talking to a provider that shared aspects of their identity and experience. Many highlighted aspects of provider communication that made them feel satisfied, such as a provider who spent a lot of time listening to their needs. Participant T (36 years old, Black/African American, queer) shared a desire for a provider who understood how their body works and how their provider interpreted having a predisposition for hypertension in the context of the shared trauma of slavery, saying:

But I guess being a practitioner of color and knowing how our bodies react and how—we’re salt-sensitive cause we came on the boats we had to hold our water and so y’know having that knowledge.

### Identity Validation and Disclosure: Negative Care

Participants described how their intersecting identities affected care experiences. One participant (V, 25 years old, Latina, queer), described how they felt when they were asked by providers about their race and ethnicity:

[…] because race and ethnicity is a question that I’ve been asked my whole life. “What is your race?” No. Matter of fact, people don’t even ask me that way. That’s what a doctor will ask me. Or “what’s your ethnicity?” [...] and it would just automatically throw me off guard […] It’s probably once in a blue moon that I would get someone just—we’ll be in an actual conversation, and they’ll ask me, and I’ll be like, “Oh, OK. I’m Spanish. I’m Dominican, Puerto Rican, and Guyanese. I’m Black and Spanish.” But I get that all the time, where it’s just something I feel like I don’t need to be told. I feel like people automatically know that I’m Spanish because of the way my hair looks or by the way my skin tone is.

Participants also described how heteronormative culture and religious values can influence their disclosure of sexual orientation and their perception of being discriminating against by healthcare providers. One participant (H, 41 years old, White, Jewish, lesbian) shared their feelings of fear around sexual identity disclosure in the context of their religious and cultural experiences:

I’m not out to any of my healthcare providers. I have two healthcare providers who are Orthodox Jewish so—and I’m Jewish […]. I don’t think [the provider] would deny me if they knew but it’s something where I worry, like would it affect my care, would they not be as nice to me? It’s something that I think about, so I’m not out to them.

Another participant (Y, 40 years old, Asian/Pacific Islander, lesbian), described how being closeted, experiencing heterosexist assumptions, and having providers not asking them about sexual orientation and behavior contributes to stressful patient–provider interactions:

Well, I mean, being a minority gay woman in this country, it’s hard to get ahead….they don't know I'm gay—they all think that oh I should have—two or three kids by now and—I don't have to work, and I should have a husband supporting me and the family… it just makes me more stressed out. Oh, [I] never, well [my provider] never asks, I don’t volunteer to tell anybody [about disclosing sexual orientation].

Some participants described perceived whiteness as an advantage that could lead to better healthcare experiences. Participant C (30 years old, Latina, lesbian/queer) discussed the absence of experiencing discrimination by providers in relation to their race, ethnicity, and skin color. They attributed their lack of discrimination experiences to their ability to pass as White, saying:

I pass off as somebody who’s White so they’re not really asking me anything [biased] or...I’m not being discriminated against—because I don’t pass as someone who is Latino.

Additionally, most participants under 21 years of age were accompanied by their parents to healthcare appointments and expressed that they did not have a safe space to discuss sexuality without a parent in the room. These participants expressed fear that their parents could find out about their sexuality, which limited the information they were able to share with providers. One participant (W, 20 years old, Black/African American, queer) recalled a difficult healthcare experience:

Only thing was just my mom being in the room so it’s not like I could ask my doctor about—you know things like sexual life and stuff like that, because my mom doesn’t agree to me being gay so it’s like if I ask those questions in front of her … she was uncomfortable—so I didn’t want to—make any strain or what not, so I just kind of kept it to myself.

Several participants described either personally having negative experiences due to presenting as masculine or knowing someone who presented as masculine that experienced mistreatment by a provider. One participant (A, 58 years old, Black/African American, lesbian) described making the conscious decision to present in more feminine ways in healthcare environments to minimize experiences of stigma:

If I’m going to doctors that I don’t, I’m not familiar with, and I can pick up that thing, that unspoken thing that we just don’t talk about, then I will swing more towards my more women’s wear for the appointment. […] I do whatever is going to get me what I need taken care of and I don’t allow the more masculine appearance side to interfere and make a person harder to work with.

Participant A described switching their gender expression and presentation from masculine to feminine so not to compromise the quality of care they may receive from a provider they perceive to be not affirming of their gender expression.

### Having LGBTQ-Knowledgeable Care Providers: Positive Care

Having LGBTQ-knowledgeable providers refer to experiences where participants’ providers knew about their LGBTQ identity and described having providers who had or did not have the subject matter expertise on the healthcare needs of LGBTQ people. When asked about what would make their healthcare experiences positive, almost all the participants emphasized the desire to have providers who are knowledgeable on WSW-specific care. Participants described how having an environment that was affirming of LGBTQ patients was essential to facilitating access to care. Many participants “came out” to their providers and felt comfortable disclosing their sexual orientation and sexual behavior to their providers when relevant to their care.

Gender-affirming care was an important determinant for transgender and non-binary participants, such as by the provider asking about the pronouns they used or the terms they used to describe their anatomy. One participant (F, 37 years old, White, sapiosexual, transgender) shared:

I’m very much—really happy with what I have because my primary [care provider] is knowledgeable in trans health, knowledgeable in trans medicine—like has ideas how to handle it and it’s great, and it’s wonderful it’s like—if more healthcare providers are actually geared towards trans medicine, understand what trans medicine is and understanding the components that go with that.

Another participant emphasized the importance of disclosing their identity so that they could receive complete and informed LGBTQ health care from their provider.

Another participant (M, 49 years old, Latina, bisexual) shared:

[…] I might want children at one point, so I want to ask him his opinion. […] I think it’s good to open the bag of worms, ‘cause it’s good for the doctor to find out about the patient overall, you know, who I *am*, because now I might be at this [provider] for, you know, osteoporosis, eye issues, so if I’m a WSW, the doctor should know the whole person and not one little segment.

### Having LGBTQ-Knowledgeable Care Providers: Negative Care

Participants described not feeling comfortable discussing topics such as their sexual health or mental health with any provider not knowledgeable on WSW-specific care. Many participants described how they have felt dismissed by their providers when they communicated their sexual health needs, even if they were “out” to their providers. Some participants shared that their providers gave unsolicited and upsetting advice, such as wrongly attributing the cause of participant visits to being overweight without looking into other health concerns. In some instances, providers presumed and focused on pregnancy risk without looking into other health concerns.

Some participants who did disclose their sexual orientation to OB/GYNs described how some providers expressed discomfort in talking about sexual health, either through outward prejudicial statements or through perceived uncomfortable body language.

Several participants described how providers often assumed they had sex with cisgender men. They emphasized that sexual orientation was assumed or ignored by providers due to non-inclusive intake forms and patient records that do not document a patient’s sexual orientation, sexual behavior, or gender identity. When asked about the last time a provider took a sexual history, many participants could not recall the last time or stated that they never had a provider ask about their sexual experience. Some participants believed they were at reduced risk of STIs primarily based on the gender of their sexual partners, which reduced their interest in sexual health screening, similarly to some provider perceptions. Many participants reported that their providers often assumed that participants were at risk of pregnancy if they were having sex, assuming sex to be penile-vaginal. One participant (*N*, 25 years old, Latina, queer) described the difficulty of finding a provider experienced with prenatal care and serving queer women, saying:

When it comes to being pregnant, it’s hard to find a provider that’s in the LGBTQ community that’s doing prenatal care […] and even if you was to sit back and get pregnant by a man or a woman, or just be getting an insemination, finding prenatal care within the LGBT community is very hard.

When asked if a provider had ever asked about their desired fertility care, almost all participants reported never having a conversation with their providers about their desire to become pregnant and have children, or to not have children in any care setting. Rather, participants recalled providers who encouraged them into birth control without asking about their sexual practices or need for birth control. Some participants wanted birth control but worried they would encounter provider resistance because they reported having sex with women.

Transgender and non-binary participants also did not feel comfortable discussing gender-related care with providers who were not knowledgeable or affirming. Participants needed to educate their providers about their sexual behavior, especially the misconception that WSW are not at risk of STIs. Participants not only described providers lacking general LGBTQ knowledge, but also described how specific aspects of their identity and gender presentation were misunderstood or disrespected by providers.

One participant (G, 22 years old, White, lesbian, non-binary) described how providers are not inclusive of transgender and non-binary people in the way they talk about anatomy, saying:

I don’t go to the doctor enough, but I do—dislike—how in the medical field—when we’re talking about female health, they generally mean, you know a vagina, and that’s you know not always true, and I don’t like being thought of as female because of my anatomy. To me those things are not related, and I don’t like when doctors talk about them as if they’re connected at all. I’d rather just use I guess anatomy terminology and not hear words such as male or female in reference to such things. Just stick with straight anatomy.

Another participant (S, 30 years old, White, lesbian) described their provider’s expressions of discomfort as a sign that providers are unequipped to work with queer patients:

Going particularly into women’s health care environments, I am visibly different, and I have experienced people who look surprised when I walk in. Often health care providers have really insufficient training on how to help queer women and again I think that impacts care.

Participant D (18 years old, Asian/Pacific Islander, lesbian) described one of their worst healthcare experiences when they sought out a gynecologist who shared their Indian identity to feel more comfortable discussing sexual health specific to WSW; but the provider’s heteronormative assumptions negatively affected their experience:

I was really excited cause I thought being a Indian Punjabi woman, I’ve grown up in a space where we don’t really talk about my health and periods are kind of stigmatized and rape culture is very prevalent in very subtle ways, so I was just hoping that this would be a [health care] space where I could you know properly learn about my health and my vagina [...] but it just ended up going back to “Have you ever had sex with a guy? I told her I have been penetrated because fingering is technically penetration as well, but I guess that just wasn’t valid for her.

This quote demonstrates how WSW can grow up in cultures where sexuality was stigmatized and then must navigate provider lack of LGBTQ health competency. This participant felt dismissed after sharing honest details of their sex life with a provider.

### Trauma-Informed Care and Bodily Autonomy with Providers: Positive Care

Participants preferred when providers were sensitive to issues around physical touch and asked for consent and explained procedures and examinations before conducting them. Participant J (*J*, 26 years old, White, bisexual/queer) shared:

I told [primary care provider] about the fact that I have a sexual assault history, and she was just so so so so so wonderful about it—she was like “Okay, we’re gonna only do the super bare minimum of stuff,” and, “if you get uncomfortable let me know, we can pause, we can stop, while this is important to your health it’s not as if it’s going to traumatize you that’s not helpful also,” you know…. I was also just so thankful and grateful for the fact that she really took the time to see me as a person and not just like, a set of body parts…

Another participant (B, 33 years old, Black/African American, bisexual) mentioned how they formed a positive relationship with their provider because the provider did not make assumptions about their family and treated them as a human being, saying,

…for people like me who don’t have family and have had traumatic experiences I mean, when you’re allowing someone to like look at your body and kind of break you apart and you know dissect all that stuff, it’s important to have someone who doesn’t just see you like—the sum of your parts, you know. It’s about someone who sees you as a human being and being treated that way.

Participants were asked to provide recommendations on how clinics could improve the care that WSW receive. Participant M (49 years old, Latina, bisexual) recommended:

[…] there should be more education with doctors, and for the receptionists of doctors […] I think doctors should bring up, you know, sexual health, more often—you know, [they do not] because the time limit, and now they want fees, they want money more, they [should ask about] how many partners do you have? Or maybe, [what kind of] family supports that you have, because I guess family is good support system […] or—what do you do if you’re, you know, stressed out?

Participants opted for a more holistic approach to health and healing rather than just treating illnesses and disease and sought alternatives to “western” health care. These WSW of color preferred to engage in care with other people of color and healing strategies beyond traditional western medicine. Participants also described self-screening such as breast self-examinations and taking initiative to screen for HIV/STIs. These practices are an alternative to and a limitation to clinical care, as this made all the participants feel more proactive about their health and in taking initiative to health and wellness that “western medicine” had failed to provide them.

Participants described seeking wellness such as acupuncture, massage therapy, meditation, and traditional and herbal medicine. One participant (*E*, 36 years old, Black/African American, non-binary and queer, woman) shared:

Everybody’s [in the health center] a TGNC POC (transgender/gender nonconforming person of color) people and a lot of them are healers in the community […] they have reiki trainings, safety trainings, meditation, guided meditation, and mindfulness practice. They have a whole bunch of stuff that can be used, you know, to bring you back to a place better than where you were originally.

Another participant (*X*, 28 years old, Asian/Pacific Islander, lesbian, bisexual and queer) shared their preference for Traditional Chinese medicine to western health care for reproductive health issues, saying:

[…] so, for me if I conjure the menstrual cramp, I would definitely go for Chinese medicine. I think they […] put really a lot of attention on the menstrual cramp, […] not just taking the pain-killing pill.

### Trauma-Informed Care and Bodily Autonomy with Providers: Negative Care

When trauma-informed care was not implemented, or patients were not given autonomy over their own bodies or respected in certain healthcare experiences, participants described feeling discomfort with their providers or healthcare settings. Such negligence in these approaches influenced the participant’s likelihood to not return to that provider or setting again. Many participants described fear of identity disclosure, fear of receiving judgment, or difficulty accessing care due to anticipated stigma they might experience from a healthcare setting with regards to their history of trauma or need for bodily autonomy in healthcare interactions. Participants also felt dismissed or belittled when they expressed to providers that they were in pain and attributed their treatment to gender-related bias.

Participants felt that some providers were not sensitive to possible patient trauma and bodily autonomy during physical or pelvic exams, or Pap smears. This issue was expressed by a participant who was a sexual assault survivor, who recommended providers screen patients for histories or experiences of sexual assault. One participant (*B*, 33 years old, Black/African American, bisexual) shared their experiences with provider stigma in the context of identity disclosure and validation, saying,

…the nurse practitioner that saw me one evening—she thought that I was kind of confused and I was doing the y’know gay till graduation thing and I was just like so over it, it was just like, ok, can you just prescribe me birth control or whatever you’re gonna do…as far as my sexuality, there have only been a handful of situations where I felt like [providers] treated me differently, and sometimes they were like ‘oh, you’re gay because you were—y’know uh—sexually assaulted.

This quote highlights the stereotype of how queer identities are invalidated when providers think they are temporary, phases, or invalid identities (e.g., gay until graduation), and the feeling invalidation of identity was indicative of a trauma-inducing space for patients. This quote sheds light on the lack of sensitivity and homophobic bias and attitudes that contribute to disengagement to health care. This also reflects how both sexual trauma and heterosexism are contributors to stigma in health care.

## Discussion

Our analysis elucidated several themes about positive and negative aspects of provider-patient engagement that affect WSW in New York City, with consideration to intersecting identities and systems of power and oppression. Participants identified positive and negative experiences related to identity disclosure, provider LGBTQ knowledge or lack thereof, and provider approaches to care. Participants described making compromises in health care when navigating challenges related to their intersecting experiences of sexism, racism, and heterosexism. Further, participants described a desire for providers who are sensitive to their bodies and listened to their concerns in a supportive and knowledgeable manner. Participants have sought out welcoming environments, such as LGBTQ health centers with inclusive intake forms, and expressed a preference for providers who were LGBTQ or shared the same racial and/or ethnic identity.

Our findings are consistent with previous studies which have demonstrated that patients desire providers who share their racial and gender identities, experience heteronormative assumptions from providers, and face challenges navigating identity disclosure (Agénor et al., 2015; Baldwin et al., 2017; Hiestand et al., 2007; Howard et al., 2019; LaVaccare et al., 2018). WSW of color report experiencing discrimination within both their racial/ethnic and LGBTQ communities, and outside of their racial/ethnic and LGBTQ communities, which can shape preferences for having a medical provider who identifies with or has experience serving people of their ethnicity and gender and sexual identity (LaVaccare et al., 2018).

In this study, we aimed to contribute to the limited literature on the healthcare experiences of people who identify as WSW and to closely examine the health inequities and health injustices facing WSW who hold multiple intersecting identities that are marginalized by a white-dominant and heteronormative healthcare system and society. Asking a diverse sample of WSW participants about their healthcare experiences in the context of their perceptions of their own salient identities allowed for nuanced descriptions of the ways individuals with different experiences of marginalization are treated by healthcare providers and the healthcare system. This study adds to the growing literature applying intersectional perspectives to LGBTQ health research (e.g., Bowleg, 2008; Mink et al., 2014; Ng, 2016) and suggests that participant self-definition of important identities may facilitate participant agency in sharing from their positionality and perspective.

This study demonstrates the need for future research on the healthcare experiences of those who identify as WSW, particularly for WSW who have gender, racial, and ethnic identities or gender expression that are stigmatized or underrepresented within healthcare research and healthcare practice amongst researchers and providers alike. Much of the research and practice aimed at improving LGBTQ care centers the experiences of those who are relatively privileged (e.g., White, able-bodied, economically privileged). This generalization of patient need and practice through this lens can risk further perpetuation of deeper institutional issues of colorism, anti-blackness, transphobia, homophobia, and xenophobia that continue to exist within the US healthcare system (Brenick et al., 2017; LaVaccare et al., 2018; Li et al., 2015).

### Strengths and Limitations

There were several key limitations in the study. First, a convenience sample of 30 WSW in New York City was not representative of the multitude and diversity of identities that exist within this population, nor was it generalizable to other jurisdictions where different geographical or cultural factors may contribute to patient experiences. Further, there was only one transgender participant in the sample, and thus, these results may not reflect the experiences of all transgender women who have sex with women. Half of the sample was recruited via social media, and the other half was recruited at LGBTQ-centered venues, which are likely frequented by a subsample of WSW individuals. Second, there may have been recall bias, as participants were asked to discuss their healthcare experiences retroactively. Third, though many participants discussed systemic barriers to adequate health care (e.g., inability to obtain health insurance, long travel and wait times to access care) the present analysis focused on patient–provider interactions. Thus, this analysis more closely informs provider-patient relations than addressing the systemic barriers and facilitators to care in New York City. It is essential that systematic approaches be employed to address issues of bias and discrimination, not only at the patient–provider level but the structural level as well.

There are several key strengths of the present study. In addition to the clinical implications described below, the qualitative method, open-ended questions, and attention to identities allowed WSW participants to share novel insights about their healthcare experiences. Patient identities informed their experiences with providers, desired care, and recommendations for improving WSW health care. For example, WSW of color described how their experiences in health care were affected by their concerns about and experiences with racism, heterosexism, and provider assumptions about gender, and reported preferring receiving care from providers that shared important identities. WSW of color had increased difficulty finding providers that both shared their racial identities and were affirming of their sexuality. Further, gender expression and presentation played a role in treatment of WSW in health care, with masculine-of-center participants making connections between their gender presentation and experiencing or anticipating bias from providers.

Half of the sample reported an income less than $20,000 per year. While our sample was not representative of the overall WSW population (New York City Department of Health and Mental Hygiene, 2017), considering that many of the insights from the present study come from low-income participants, it is important that the suggestions for patient–provider relations from this study be employed in healthcare facilities that service Medicaid patients and uninsured individuals.

### Clinical Implications and Contributions

As participants reported more positive experiences with providers who shared their identities and limited access to these providers, increasing the representation of LGBTQ healthcare providers of color would improve access to health care. Systemic racism contributes to inequities in education quality and access for many people of color (Merolla & Jackson, 2019), biased medical school admissions (Lucey & Saguil, 2020), and many other forms of discrimination that culminate in a physician workforce that is mostly White (56.2%) and mostly made up of men (64.1%) (AAMC, 2019). The Association of American Medical Colleges (AAMC) 2019 executive summary of diversity in medicine does not report the sexuality of providers and only assumes a binary of women and men when reporting on gender of its providers (AAMC, 2019). Both are demonstrative of the lack of attention to the importance of a comprehensive understanding of gender and sexual identity in the medical field and the need for a survey that captures the gender and sexualities of healthcare providers in AAMC and beyond.

A second clinical implication was that providers should take a trauma-informed and patient-centered approach to care that seeks to an understand the discrimination experienced by WSW in health care and other past traumas that may influence their access to care (e.g., sexual assault, obstetric violence, medical violence, etc.). Providers need training on how to take a non-judgmental approach to care, including asking open-ended questions to allow patients to self-define their own identities and experiences (Klein & Nakhai, 2016). Further, there should be increased training on LGBTQ health needs and experiences for providers which include concerns specific to WSW, as many of our participants felt their experiences had been overlooked. Providers who are more knowledgeable about WSW sexual behavior and sexual health needs may avoid making cis and heteronormative assumptions, such as assuming all sex involves penile–vaginal intercourse. Increased provider knowledge and practice engaging patients in open conversations about sexual health may enable patients to define their sexual orientation and sexual behaviors for themselves and experience a higher quality of care. Making healthcare spaces and providers more culturally sensitive to LGBTQ health experiences can increase LGBTQ individuals’ comfort in healthcare settings and willingness to engage in care. In New York State, patients less than 18 years of age have a right to confidential sexual and reproductive healthcare, and providers should ask parents or caregivers to step out of the room to have these conversations if they accompany patients.

As of spring 2022, the NYC Health Map, a search engine designed to help patients find the healthcare services they need in New York City, listed 83 healthcare sites listed that provide primary care which are deemed “LGBTQ-knowledgeable” (New York City Department of Health and Mental Hygiene, 2022). While this is a great step in improving LGBTQ health care, the criteria for this label are often unclear or lack nuance as to the specific factors that affect LGBTQ healthcare experiences. Researchers and healthcare professionals in New York City and other jurisdictions should develop a clear set of criteria for what makes a clinic LGBTQ-knowledgeable, regularly update the criteria with increasing research, and regularly assess clinics so that LGBTQ individuals can have more confidence in the potential to receive experienced and affirming care in those settings. The narratives from this study demonstrate the importance of considering the role of gender expression, not just gender identity or sexual orientation, as an important factor that overlaps with other experiences to impact healthcare experiences, including discrimination. Further, several of the key findings from this study relate to the importance of providers having awareness of the effect of trauma histories with regard to racism, sexism, homophobia, transphobia, sexual assault, and other forms of mistreatment.

### Consequences of Discrimination and Trauma

There is a lack of public health research and discourse that accounts for the experiences of those who identify as WSW and as women of color. Furthermore, healthcare providers are not creating adequate settings for this population to receive competent care, which can undermine healthcare engagement and well-being (e.g., Brenick et al., 2017). Inadequate care for WSW also contributes to many WSW consciously choosing to not disclose their sexual orientation or sexual behaviors to healthcare providers when relevant to their care out of fear of stigma, discrimination, and mistreatment.

Participants reported experiences where their needs and wants were not respected, where they felt physically uncomfortable or violated by providers, or where past traumas affected their experiences receiving exams. Thus, the present research supports the importance of providers taking trauma seriously and learning how to provide trauma-informed care, a practice that acknowledges the impact of trauma on well-being (Coston, 2021; Raja et al., 2015). Trauma-informed care requires healthcare providers to engage in compassionate care approaches in their interactions with clients with the understanding that all people can come from a history of trauma and abuse (Coston, 2021). Considering a trauma-informed approach is essential when taking an intersectional perspective because overlapping systems of oppression contribute to the prevalence of traumatic experiences, both of stigma and discrimination and other forms of trauma, such as sexual assault (e.g., Coulter et al., 2017). Experiences of trauma both in health care and outside of health care can affect the needs and experiences of patients in care (e.g., Palmieri & Valentine, 2021), and thus, trauma is important to consider when exploring the experiences of diverse WSW.

### Conclusion

This present study aims to contribute to the limited literature on the healthcare experiences of those who identify as WSW to better understand experiences in patient care that may contribute to health disparities, with consideration to intersecting identities rooted in reinforced systems of power and oppression. This study offers suggestions for both providers and patients to how health care is practiced, considered, and received amongst the LGBTQ population, specifically centering those who identify as WSW.

In the present study, we asked participants directly to self-define the identities in the order that felt most important to them and elucidate how these identity experiences and intersections impacted their experiences in health care. In examining participant experiences across a range of experiences, we were able to identify patterns that both apply across groups and those that are uniquely situated in a particular experience. For example, there was a pattern that women of color were more likely to name their race and ethnicity as important identities, whereas White participants more often did not mention their race. This may be due to White participants having the privilege and perception of not having to think about their own race as compromising their interactions when seeking health care. This identifies an area for future exploration. Further, while individuals across racial and ethnic communities indicated preferences for providers that shared their racial identity, there were also differences. For example, one Black participant specifically discussed slavery, a Chinese immigrant participant compared healthcare cultures in the USA and China, and a Punjabi Indian woman described feeling that she lived a “double life.” Each participant’s experience of their own identities, histories, and cultures uniquely affected their experiences of health and relationship to healthcare providers, with this study demonstrating the wide range of experiences even among a small group of WSW participants. To provide truly supportive care for WSW, providers must consider the way each patient’s experiences occur in the context of intersecting systems of power and oppression.

While the findings presented here reinforce conclusions from the existing literature, they also extend beyond by allowing participants to self-define their own important identities and demonstrate patterns of similarity and difference between experiences of marginalization for exploration in future research. These patterns suggest to providers the importance of increasing their knowledge about LGBTQ healthcare needs and engaging in patient-centered models of care that center each individual patient in the context of their important identities. Future studies should consider deeper description and analysis of the differing experiences and nuances of how WSW participants’ racial, ethnic, sexual, and gender identities impact their healthcare treatment and experiences. This will allow providers to break the generalizability of patient treatment based on mere archetypes of identities. Providers can then feel encouraged to collaborate to create patient treatment alongside patients that are attuned and responsive to the patient’s individual experiences and unique needs. This can inspire care that ultimately affirms a patient’s identity through the very process of their collaborative care being constructed, instead of care being rote or restricted by the assumptions the provider makes of the patient’s identity.

This study exhibits the critical role of patients’ intersecting identities in their relationships with providers. Honoring and centering the identities that inform patient experiences in health care, research, and society is a necessary step toward the development of intervention and advocacy to further advance health equity and justice.

## Data Availability

Not applicable.
